# Antipsychotic Potential of Opioids: Rethinking Substance-Induced Psychosis and Treatment Stratification

**DOI:** 10.3390/jcm14155596

**Published:** 2025-08-07

**Authors:** Angelo G. I. Maremmani, Filippo Della Rocca, Silvia Bacciardi, Manuel Glauco Carbone, Icro Maremmani

**Affiliations:** 1 UniCamillus, International Medical University in Rome, Via di Sant’Alessandro 8, 00131 Rome, Italy; filippo.dellarocca@yahoo.it (F.D.R.); s.baciard@gmail.com (S.B.); manuelglaucocarbone@gmail.com (M.G.C.); icromaremmani@gmail.com (I.M.); 2 VP Dole Research Group, PISA-School of Addiction Medicine, G. De Lisio Institute of Behavioural Sciences, Via di Pratale 3, 56121 Pisa, Italy; 3 Addiction Unit, Department of Mental Health and Addictions, ASL5 Liguria NHS, Via Dalmazia 1, 19124 La Spezia, Italy; 4 Department of Psychiatry and Addictions, Section of Psychiatry, North-Western Tuscany Local Health Unit, Tuscany NHS, Versilia Zone, Via Aurelia 335, 55041 Lido di Camaiore, Italy; 5 Division of Psychiatry, Department of Medicine and Surgery, University of Insubria, Viale Luigi Borri 57, 21100 Varese, Italy

**Keywords:** substance-induced psychosis, opioids, methadone, buprenorphine, antipsychotics, withdrawal, dual diagnosis

## Abstract

Substance-induced psychosis is a recognized clinical entity, commonly linked to cannabinoids, stimulants, hallucinogens, alcohol, and polysubstance use. These agents may provoke transient or persistent psychotic symptoms during intoxication or withdrawal. Opioids, however, constitute a noteworthy exception: psychosis is rarely observed during opioid intoxication, and emerging data suggest that opioid agonists might even exert antipsychotic-like effects. This article examines the paradoxical interaction between opioids and psychosis, with attention to clinical reports of psychotic symptoms arising following abrupt discontinuation of methadone or buprenorphine. In numerous cases, symptoms resolved swiftly after reintroduction of the opioid agonist, implying a neuromodulatory role. Opioids, unlike other substances of abuse, seem to lack intrinsic psychotogenic effects and may influence dopaminergic activity via kappa-opioid receptor antagonism and endorphinergic mechanisms. This challenges standard models of substance-induced psychosis and calls for a refined understanding of opioid pharmacodynamics in psychiatric contexts. In psychotic presentations among polysubstance users who also use opioids, restoring opioid agonist therapy should be prioritized, with antipsychotics reserved as second-line options—preferably agents with favorable receptor profiles. Where opioids are not involved, antipsychotics remain first-line, but should be applied judiciously, with efforts to taper when clinically appropriate.

## 1. Introduction

Dual disorders are a prevalent and complex clinical reality, particularly in psychiatric and addiction care settings. In recent years, both clinical practice and scientific research have contributed to a deeper understanding of the intricate relationship between substance use and psychosis [1]. Psychotic symptoms in this context can generally be classified into three categories: (i) substance-related paranoid ideation, (ii) intoxication-induced psychosis, and (iii) primary psychotic disorders that occur independently of substance use (i.e., dual diagnosis). Substance-induced psychosis is a well-documented phenomenon, especially among individuals with histories of stimulant, cannabis, hallucinogen, or polysubstance use. Studies indicate that up to 40% of individuals with methamphetamine dependence and around 20% of chronic cannabis users may develop psychotic symptoms that are often indistinguishable from primary psychotic disorders [2,3]. These episodes may occur either during intoxication or withdrawal, complicating both diagnosis and treatment, especially if associated with substances such as methamphetamine, amphetamine, cannabis, cocaine, alcohol, lysergic acid diethylamide (LSD), phencyclidine (PCP), ecstasy, and ketamine [4]. In contrast, opioids appear to represent a pharmacological exception. Evidence for opioid-induced psychosis is limited and inconsistent, and unlike most other substances of abuse, opioids rarely induce psychotic symptoms during intoxication [5]. Moreover, recent clinical observations suggest that opioid agonists such as methadone and buprenorphine may exert stabilizing effects on psychosis, particularly when used as part of long-term maintenance therapy. This article proposes a specific clinical perspective on the so-called “opioid exception,” exploring the possible neurobiological mechanisms underlying this phenomenon and discussing its implications for antipsychotic treatment strategies in dual diagnosis populations.

This perspective is based on a narrative review of relevant literature identified through PubMed and Scopus searches using the terms “opioid withdrawal psychosis”, “opioid-induced psychosis”, “methadone and psychosis”, and “buprenorphine and psychosis”. Clinical studies, case reports, case series, and historical reviews were included, with no restriction on publication date. Selected references were integrated with clinical experience and theoretical considerations from the authors’ research background.

## 2. The Opioid Exception in Substance-Induced Psychosis

As previously noted, psychotic symptoms during intoxication are frequently observed in association with substances such as cannabinoids, stimulants, hallucinogens, alcohol, and various combinations of drugs. In contrast, opioids are unique in that they generally lack direct psychotogenic properties [5]. However, a growing body of literature has highlighted a distinct and paradoxical phenomenon: the emergence of psychotic symptoms following the abrupt discontinuation of opioid agonists. Whether these episodes represent a rare variant of withdrawal-related psychosis or reflect the unmasking of an underlying psychotic disorder previously suppressed by opioid agonist therapy remains an open question. A recent review by Lozano-Lopez and colleagues—including case reports, case series, clinical studies, and narrative analyses—found that psychotic symptoms typically appeared within days after discontinuing opioid treatment. Importantly, in most of these cases, reinstating the opioid agonist led to significant clinical improvement and full remission of psychotic features [6].

## 3. Psychosis Associated with Methadone Withdrawal

Methadone withdrawal typically presents as a classical opioid withdrawal syndrome, characterized by symptoms such as restlessness, pupillary dilation, sweating, insomnia, irritability, sneezing, nausea, vomiting, and diarrhea. Psychosis is not generally included among these manifestations. However, a growing number of case reports suggest that methadone withdrawal may, in rare instances, be associated with acute psychotic episodes.

Pinho and colleagues recently described the case of a 43-year-old woman with a history of long-term methadone use who developed a first episode of psychosis during methadone down-titration. She exhibited persecutory delusions and auditory hallucinations in the absence of classical withdrawal symptoms. Medical evaluations were unremarkable. The patient was diagnosed with first-episode psychosis and was involuntarily admitted to a psychiatric hospital. Due to a history of hormone-sensitive breast cancer and the development of paliperidone-induced hyperprolactinemia, treatment was switched to aripiprazole, a prolactin-sparing antipsychotic. Eventually, psychotic symptoms resolved within six weeks without reintroduction of methadone. It remains uncertain whether this case reflects a rare manifestation of opioid withdrawal or whether methadone had previously exerted a protective effect by masking an underlying psychotic disorder [7].

The Spanish group led by Lozano-Lopez emphasized that opioid detoxification may unmask latent psychotic vulnerability, especially in individuals at high risk of developing chronic psychotic disorders or schizophrenia. Their review included seven reports demonstrating a temporal association between opioid withdrawal and the onset of psychotic symptoms, with marked clinical improvement following reintroduction of the opioid agonist. According to the authors, the sedative and mood-regulating properties of opioids, mediated by the endogenous opioid system, may partly explain this phenomenon and suggest a possible antipsychotic effect [6].

An additional mechanistic hypothesis was proposed by Cobo and colleagues, who reported two cases of typical psychosis triggered by methadone withdrawal. Both patients had no personal or family history of psychotic disorders. Notably, neither case presented with a typical withdrawal syndrome, and psychotic symptoms emerged in close temporal proximity to the abrupt cessation or rapid reduction of methadone maintenance therapy. The authors suggested that disruption in central opioid/dopamine neuromodulation may underlie this clinical presentation [8].

A more recent case from the United States further contributes to the literature. A patient who abruptly discontinued methadone treatment was initially managed with aripiprazole and hydroxyzine for persistent paranoid ideation and anxiety. Despite pharmacological treatment, symptoms remained unresolved for 17 days, during which the patient refused to resume methadone. On day 18, she agreed to restart methadone at 10 mg daily, and hydroxyzine was discontinued. Within 48 h, she reported improvements in anxiety and a gradual remission of paranoid ideation [9].

Going back several decades, Levinson and colleagues documented new-onset psychosis in four patients undergoing methadone tapering. Two of the patients had no prior history of psychosis; one had experienced a psychotic episode over 20 years earlier, and another had a previous schizophrenia diagnosis but remained asymptomatic for over a year while treated exclusively with methadone. In one case, psychosis resolved spontaneously, while others required neuroleptic intervention. Notably, in one case, symptom resolution occurred only after methadone was reinstated. None of the cases exhibited the classical features of methadone withdrawal [10].

The antipsychotic effect of methadone has also been described anecdotally. In a historical report by Brizer and colleagues, methadone was administered at low doses (approximately 25 mg/day), in addition to neuroleptics, in patients with treatment-resistant chronic paranoid schizophrenia, producing significant clinical improvement. Notably, none of the patients had a history of substance use or dependence [11].

## 4. Psychosis Associated with Buprenorphine Withdrawal

Similar cases have been reported in relation to buprenorphine discontinuation.

Lin and colleagues described the onset of psychotic symptoms in a 41-year-old woman with a history of bipolar disorder who developed persecutory delusions and hallucinations shortly after abruptly discontinuing buprenorphine. Notably, the patient had no prior personal or family history of psychotic disorders [12]. This report aligns with a growing body of similar case studies.

Navkhare presented the case of a 40-year-old man with no previous psychiatric history or concurrent substance use—except for opioids—who developed persecutory delusions accompanied by visual hallucinations following abrupt cessation of buprenorphine. Antipsychotic treatment with olanzapine was only partially effective and eventually discontinued. Upon reinitiating buprenorphine at 2 mg twice daily, the patient experienced complete remission of psychotic symptoms within 48 h. Remarkably, no recurrence was observed over the following six months while the patient remained on a tapering regimen of 4 mg/day [13].

Other reports have reinforced this temporal association and highlighted the importance of the mode of discontinuation. Weibel and colleagues emphasized that abrupt versus gradual tapering may play a critical role in the emergence of psychotic symptoms [14].

Karila and colleagues reported a case involving a 32-year-old man undergoing buprenorphine withdrawal. He had been on a stable dose of 6 mg/day for one year and had a past diagnosis of psychosis treated with aripiprazole, which had been discontinued by his psychiatrist during a period of incarceration. Following a reduction in buprenorphine dosage, the patient developed psychotic symptoms that proved resistant to loxapine (up to 100 mg/day). Only upon reintroducing buprenorphine at the previous dose of 6 mg/day did the psychotic symptoms remit within 24 h. The patient remained clinically stable during a six-month follow-up while continuing buprenorphine at a consistent dose [15].

The antipsychotic effects of buprenorphine have also been described anecdotally. In a historical report by Schmauss and colleagues, 10 patients with psychotic disorders, none of whom were receiving antipsychotic treatment at baseline, exhibited clinical improvement following the initiation of buprenorphine therapy [16].

## 5. Psychosis Associated with Heroin and Other Prescription Opioid Withdrawal

Although uncommon, psychotic symptoms have also been reported following the withdrawal of illicit opioids such as heroin, as well as prescription opioid analgesics, including morphine, oxycodone, and tramadol.

Sharma and colleagues described a case of heroin withdrawal-induced psychosis in a 25-year-old man with no prior psychiatric or family history. Upon hospitalization, the patient exhibited irritability, delusions of parasitosis, and both auditory and visual hallucinations. Treatment with risperidone (3 mg/day) and low-dose buprenorphine (2 mg/day) led to clinical remission [17].

Freye and colleagues reported the case of a 57-year-old man who developed psychotic symptoms—including auditory, visual, and olfactory hallucinations, disorganized behavior, paranoid ideation, and persecutory delusions—after abruptly discontinuing tramadol, which had been prescribed for chronic back pain via an intrathecal morphine pump over several years [18]. Similarly, a large study by Senay and colleagues involving 422 patients treated with tramadol for opioid dependence reported both typical and atypical withdrawal symptoms. Atypical symptoms included hallucinations, suspiciousness, panic attacks, and delirium, in addition to classical opioid withdrawal manifestations such as anxiety, diarrhea, insomnia, lacrimation, rhinorrhea, and sweating [19]. The atypical withdrawal symptoms of tramadol, such as psychosis, may be related to its activity beyond the opioid receptors—namely, on catecholaminergic and serotonergic systems. Rajabizadeh and colleagues noted that psychosis during tramadol withdrawal typically resolves within a few days following symptomatic treatment [20]. Other studies have highlighted that tramadol withdrawal may, in some cases, be accompanied by atypical symptoms not commonly observed during opioid withdrawal—such as hallucinations, paranoia, extreme anxiety, panic attacks, confusion, and unusual sensory experiences including numbness and tingling in the extremities [19].

Casado-Espada et al. described the case of a 36-year-old man with no personal or family history of psychiatric illness, who was being treated with multiple analgesics—including oxycodone—for chronic non-inflammatory back pain. Following the abrupt discontinuation of oxycodone, he developed a fluctuating psychotic picture characterized by delusions of harm and contamination, olfactory hallucinations, and aberrant behavior. Symptoms eventually subsided after initiation of antipsychotic treatment [21].

Aiyer and colleagues presented a similar case involving a 57-year-old man with long-standing treatment for chronic back pain via an intrathecal morphine pump. The patient developed disorganized thinking, delusional thoughts, paranoia, and both auditory and visual hallucinations. Investigation revealed that the intrathecal pump had malfunctioned and was no longer delivering morphine. Psychotic symptoms rapidly improved following opioid rotation and the initiation of oral oxycodone, strongly suggesting that the psychosis was withdrawal-induced [22].

## 6. Psychosis Associated with Opioid Intoxication

There is limited research on opioid-induced psychosis, with most evidence restricted to isolated case reports. Tumenta and colleagues described the case of a 36-year-old male with a history of cannabis use and no previous psychiatric diagnosis who developed acute psychosis while receiving high doses of hydromorphone for a sickle cell pain crisis. Although the authors excluded a delirium diagnosis, they concluded that, while certain substances have a well-established link to psychosis, the association with opioids remains less clearly defined. Interestingly, hydromorphone acts as an agonist at kappa-opioid receptors, which may be relevant to its psychotropic profile [23].

Another case was reported by Dhanabalan and colleagues, involving a 67-year-old African American man with a diagnosis of “schizophrenia, unspecified”. The onset of auditory and visual hallucinations coincided with the initiation of hydrocodone 5 mg/acetaminophen 325 mg for chronic back pain. However, the patient had previously reported episodes of hallucinations and paranoia, and the psychotic symptoms in this instance were likely attributable to postictal psychosis following a seizure, within a complex clinical context that included chronic hepatitis C and a recent hepatic coma. Additionally, the patient was a recreational cannabis user and had experienced visual hallucinations [24].

## 7. Mechanistic Hypotheses: Opioid Modulation of Psychosis

Several neurobiological mechanisms may underlie the interaction between opioids and psychosis. The hypothesis that opioid agonist medications might exert antipsychotic effects dates back several decades. As early as the 1970s and 1980s, clinical observations and experimental data suggested a potential role for both exogenous and endogenous opioids in modulating psychotic symptoms. Reports included patients with schizophrenia who appeared to self-medicate with opiates [25], findings from methadone maintenance programs [26], and evidence from clinical [27] and neuroendocrine [28] studies supporting the hypothesis that methadone may possess antipsychotic properties.

Nearly 40 years ago, Brizer and colleagues conducted a placebo-controlled single-crossover study in seven patients with treatment-resistant chronic paranoid schizophrenia. Methadone, administered at low doses (approximately 25 mg/day), in addition to neuroleptics, produced significant clinical improvement. Notably, none of the patients had a history of substance use or dependence [11]. In a parallel study, Schmauss and colleagues evaluated the effects of buprenorphine (0.2 mg/day) in 10 neuroleptic-free patients with schizophrenia suffering from hallucinations, delusions, and severe formal thought disorders. Buprenorphine produced pronounced antipsychotic effects lasting about four hours, although it was ineffective in patients with predominantly residual symptoms [16].

Understanding the pharmacological basis for the potential antipsychotic properties of opioid agonists is complex. Buprenorphine acts as a partial mu-opioid receptor agonist and a kappa-opioid receptor (KOR) antagonist. Since KOR activation has been implicated in dysphoria, hallucinations, and stress-induced neurotoxicity, its antagonism may play a protective role against psychosis through modulation of the mesolimbic dopamine system [29]. Methadone, a full mu-opioid receptor agonist, may influence psychosis through endorphinergic pathways and indirect dopaminergic modulation [30]. Chronic opioid agonism is known to downregulate dopamine receptor sensitivity and alter dopaminergic tone; abrupt discontinuation may lead to a rebound dopaminergic surge, potentially contributing to psychotic decompensation [31]. This model is supported by early-phase clinical data suggesting that methadone or low-dose buprenorphine can transiently reduce positive psychotic symptoms in opioid-naïve patients with schizophrenia [11,16].

Further supporting this hypothesis, the effects of naltrexone—a μ-opioid receptor antagonist—appear to contrast with those of long-acting opioid agonists. While methadone and buprenorphine are well-established first-line treatments for opioid use disorder, naltrexone is typically considered a second-line therapy, potentially suitable only for select subgroups of patients. A recent study by Lone and colleagues reported that treatment retention among patients receiving naltrexone maintenance therapy was negatively affected by several variables, including the presence of psychiatric comorbidity [32].

## 8. Clinical Observations from the Pisa School of Addiction Medicine

The Pisa School of Addiction Medicine, at the University of Pisa, has systematically explored the clinical and theoretical intersections between opioid use and psychopathology. Over the last three decades, this group has developed a coherent research line centered on the hypothesis that, for a subgroup of individuals, opioids may play a role as a self-medication tool in psychotic symptoms.

Early observational studies examined the use of illicit methadone among individuals seeking their first opioid agonist treatment. In one such study [33], heroin users who reported consuming non-prescribed methadone displayed a distinct profile: they had a higher rate of comorbid psychiatric disorders, lower daily heroin use, and less polysubstance abuse. These findings supported the idea that the use of methadone, even when not medically supervised, could represent a form of non-specific self-medication aimed at reducing internal distress or subthreshold psychotic symptoms. Further empirical support comes from a study comparing 23 heroin-dependent patients with psychotic features to 209 non-psychotic peers starting opioid agonist therapy [34]. The psychotic group, despite exhibiting more severe psychiatric symptoms, showed a milder addiction trajectory. They sought treatment earlier, had a shorter history of heroin dependence, and used fewer other substances. These characteristics were interpreted as consistent with the idea that opioid use in this subgroup was not primarily driven by craving or hedonic pursuit but rather by attempts to manage distressing cognitive and emotional experiences. A subsequent temporal priority analysis [35] further validated this model by demonstrating that psychotic or anxiety disorders often predated heroin use in dual diagnosis patients. This finding lent credibility to Khantzian’s self-medication hypothesis [26,36], which posits that substance use may originate as an attempt to alleviate pre-existing psychiatric symptoms rather than as a direct pathway to addiction. The University of Pisa Research Group also evaluated the impact of methadone dosages during psychiatric hospitalization. In a retrospective study of 114 heroin-dependent inpatients experiencing manic or psychotic episodes, those who received higher doses of methadone were significantly less likely to be prescribed additional psychotropic medications at discharge [37]. This suggested that methadone may exert a direct stabilizing effect on psychiatric symptoms beyond withdrawal and craving control. Finally, a comparative study between bipolar I and chronic psychotic heroin users [38] found that psychotic individuals had milder addiction severity and initiated opioid use earlier than their bipolar counterparts. These findings reinforced the notion that, in psychotic patients, opioids might serve as a coping mechanism for early-onset psychopathology rather than functioning solely as substances of abuse. From a clinical standpoint, the Research Group has also demonstrated the effectiveness of long-acting opioids, particularly methadone, in mitigating psychopathological symptoms beyond craving and withdrawal. Methadone has been shown to positively influence specific domains of heroin use disorder psychopathology, including traits related to sensitivity and psychoticism—reinforcing its utility as a therapeutic agent in patients with complex dual diagnoses [39].

## 9. Therapeutic Implications: A New Role for Antipsychotics in Dual Diagnosis

The recognition of an “opioid exception” has important clinical implications, necessitating a re-evaluation of treatment strategies for psychosis in individuals with substance use disorders, especially those involving opioids. In polysubstance users who include opioids, clinicians should assess whether psychotic symptoms are temporally related to opioid withdrawal. If a clear link is established, the priority should be to reintroduce or optimize opioid agonist therapy (e.g., methadone or buprenorphine) before initiating antipsychotic medication. In such cases, antipsychotics should be reserved for patients whose symptoms persist despite adequate opioid stabilization.

In general, maintaining patients with schizophrenia on antipsychotic medications after an acute episode significantly reduces the risk of relapse—from 53.2% to 15.6% over an average follow-up of 9.7 months. However, the burden of side effects is substantial: antipsychotics can cause extrapyramidal symptoms (EPSs), sedation, weight gain, hyperprolactinemia, and metabolic disturbances, and they have even been associated with sudden cardiac death [40]. For dual diagnosis patients, these risks are particularly critical, as the hedonic system is often already compromised by chronic substance use. Understanding the trajectory of addicted individuals is essential in this context. In the early stages of addiction, the brain’s reward system remains responsive to both drug-related and natural stimuli. Over time, however, prolonged substance exposure leads to hypofunction of the reward circuitry, resulting in a chronic state of hypophoria and anhedonia [41]. This so-called “hypophoric trap” [42] should be a key consideration in the use of antidopaminergic agents. Antipsychotics with a strong dopamine-blocking profile may exacerbate this condition, further impairing motivation and emotional responsiveness. Thus, in addition to traditional concerns, such as weight gain, EPSs, prolactin elevation, QTc prolongation, and sedation, clinicians should consider the potential hedonic toxicity of antipsychotics—particularly in patients with substance use disorders. This is especially relevant given real-world data showing that 20–40% of schizophrenia patients worldwide are prescribed higher-than-recommended antipsychotic doses. High-dose prescribing is more common in patients with paranoid schizophrenia, antisocial personality traits, or co-occurring substance use disorders [43].

Pharmacological treatment of psychosis in the context of substance use remains a clinical challenge. Comparative studies of antipsychotics in dual diagnosis populations are scarce and often methodologically limited. Most available data derive from case reports, open-label studies, or investigations focusing primarily on cannabis, alcohol, and stimulant use, with limited evidence concerning opioids [44].

One key pharmacodynamic concept is the dopamine D2 receptor binding affinity and dissociation kinetics of antipsychotics. First-generation (typical) antipsychotics—such as haloperidol and fluphenazine—bind tightly and dissociate slowly from the D2 receptor, resulting in high dopaminergic blockade and increased risk of EPSs. In contrast, second-generation (atypical) antipsychotics like quetiapine and clozapine exhibit rapid D2 dissociation, allowing for more physiologic dopaminergic signaling and reduced motor side effects [45]. Intermediate agents such as olanzapine and loxapine fall somewhere in between. Given this pharmacological spectrum, second-generation agents tend to be better tolerated and are generally preferred in dual diagnosis populations [44].

Nevertheless, high-quality evidence comparing specific antipsychotics in dual diagnosis patients remains limited. For example, while risperidone is widely used, no definitive conclusions can be drawn about its superiority over other agents in this population [46]. Emerging interest has focused on partial D2 agonists (third-generation antipsychotics), such as aripiprazole, brexpiprazole, and cariprazine. These compounds modulate dopamine signaling rather than fully blocking it, attenuating hyperdopaminergic activity in psychosis while simultaneously enhancing dopaminergic tone in hypodopaminergic states [44,47]. This receptor profile may be particularly well suited to patients with dual diagnosis, in whom chronic substance exposure disrupts dopaminergic homeostasis.

Beyond D2 modulation, third-generation antipsychotics exhibit a range of pharmacological actions that may be advantageous in this context. These include the following: (i) D3 antagonism or partial agonism, which may enhance motivation and cognitive function; (ii) 5-HT2A antagonism and 5-HT1A partial agonism, associated with anxiolytic and antidepressant effects; and (iii) 5-HT7 antagonism, implicated in mood and cognitive regulation [48,49].

Despite their favorable pharmacodynamic profiles, partial D2 agonists have not yet demonstrated robust superiority in randomized trials. The notable exception is clozapine, which remains the most effective antipsychotic in treatment-resistant schizophrenia. Recent data suggest that clozapine may also confer benefits in patients with comorbid substance use. A study from the University of British Columbia found that clozapine treatment was associated with a significantly lower rate of methamphetamine relapse and a higher likelihood of sustained abstinence compared to other antipsychotics. These findings remained significant even when evaluating any type of substance use relapse [50,51]. Clozapine should also be considered in patients with schizophrenia and comorbid substance use, as it appears to offer superior protection against relapse compared to other antipsychotics [52].

Given the complexity of treating psychosis in the context of addiction, clinicians should adopt a recovery-oriented approach that prioritizes functional outcomes and quality of life. Fast D2 dissociation as well as the latest antipsychotics generation—with their dopaminergic modulation and reduced hedonic suppression—may offer a therapeutic advantage. In stabilized patients, dose reduction strategies should be actively considered to minimize iatrogenic burden and support long-term recovery goals [53].

## 10. Conclusions and Recommendations

Although a definitive causal relationship between opioids and psychosis cannot be established—partly due to the lack of large-scale randomized controlled trials—clinical data reviewed above underscore a unique and clinically significant interaction. Unlike most substances of abuse, opioids appear to lack intrinsic psychotogenic properties and may, under specific conditions, exert stabilizing effects on psychotic symptoms, particularly within the framework of opioid agonist therapy.

This “opioid exception” challenges the conventional view of substance-induced psychosis as a unitary diagnostic category and emphasizes the importance of individualized treatment strategies based on the type of substance, the temporal pattern of symptom emergence, and the patient’s psychiatric history. It also calls for a reassessment of when and how antipsychotic medications should be introduced in patients with dual diagnoses.

We propose a staged clinical approach:

- In patients with opioid use and comorbid psychosis,
- Carefully assess the temporal association between opioid withdrawal and the onset of psychotic symptoms;
- Prioritize the reinstatement or optimization of opioid agonist therapy (e.g., consider switching from buprenorphine to methadone when appropriate);
- Reserve antipsychotic treatment for persistent symptoms. In adherent patients, clozapine may be considered. Otherwise, second- or third-generation antipsychotics with partial D2 agonism or rapid D2 dissociation profiles are preferable.
- In patients with primary psychotic disorders and no opioid involvement,
- Consider clozapine in compliant patients under appropriate medical supervision. Alternatively, initiate treatment with second- or third-generation antipsychotics, taking into account their receptor binding profiles and side-effect burden.

When clinically feasible, plan for gradual dose reduction and limit treatment duration to the shortest effective time.

Recognizing the opioid exception highlights the need for a translational, recovery-oriented approach that draws from addiction medicine, psychopharmacology, and personalized psychiatry. Future research—including prospective controlled trials—is crucial to validate these clinical insights and to develop evidence-based treatment algorithms tailored to this often-overlooked and complex population.

## Data Availability

No new data were created or analyzed in this study. Data sharing is not applicable to this article.
